# Economic Burden and Health Care Access for Patients With Inflammatory Bowel Diseases in China: Web-Based Survey Study

**DOI:** 10.2196/20629

**Published:** 2021-01-05

**Authors:** Qiao Yu, Chunpeng Zhu, Shuyi Feng, Liyi Xu, Shurong Hu, Hao Chen, Hanwen Chen, Sheng Yao, Xiaoying Wang, Yan Chen

**Affiliations:** 1 Department of Gastroenterology, the Second Affiliated Hospital, Zhejiang University School of Medicine Hangzhou China

**Keywords:** inflammatory bowel disease, Crohn disease, ulcerative colitis, primary care provider, emergency room, eHealth, gastroenterology, proctology

## Abstract

**Background:**

The increasing incidence of inflammatory bowel disease (IBD) has imposed heavy financial burdens for Chinese patients; however, data about their financial status and access to health care are still lacking. This information is important for informing patients with IBD about disease treatment budgets and health care strategies.

**Objective:**

The aim of this study was to evaluate the economic status and medical care access of patients with IBD through the China Crohn’s & Colitis Foundation web-based platform in China.

**Methods:**

Our study was performed in 14 IBD centers in mainland China between 2018 and 2019 through WeChat. Participants were asked to complete a 64-item web-based questionnaire. Data were collected by the Wenjuanxing survey program. We mainly focused on income and insurance status, medical costs, and access to health care providers. Respondents were stratified by income and the associations of income with medical costs and emergency visit times were analyzed.

**Results:**

In this study, 3000 patients with IBD, that is, 1922 patients with Crohn disease, 973 patients with ulcerative colitis, and 105 patients with undetermined colitis were included. During the last 12 months, the mean (SD) direct and indirect costs for per patient with IBD were approximately US $11,668.68 ($7944.44) and US $74.90 ($253.60) in China. The average reimbursement ratios for most outpatient and inpatient costs were less than 50%. However, the income of 85.5% (2565/3000) of the patients was less than ¥10,000 (US $1445) per month. Approximately 96.5% (2894/3000) of the patients were covered by health insurance, but only 24.7% (741/3000) of the patients had private commercial insurance, which has higher imbursement ratios. Nearly 98.0% (2954/3000) of the patients worried about their financial situation. Thus, 79.7% (2392/3000) of the patients with IBD tried to save money for health care and even delayed their medical treatments. About half of the respondents (1282/3000, 42.7%) had no primary care provider, and 52.2% (1567/3000) of the patients had to visit the emergency room 1-4 times per year for the treatment of their IBD. Multivariate analysis revealed that lower income (*P*=.001) and higher transportation (*P*=.004) and accommodation costs (*P*=.001) were significantly associated with the increased number of emergency visits of the patients.

**Conclusions:**

Chinese patients with IBD have enormous financial burdens and difficulties in accessing health care, which have increased their financial anxiety and inevitably influenced their disease outcomes. Early purchase of private insurance, thereby increasing the reimbursement ratio for medical expenses, and developing the use of telemedicine would be effective strategies for saving on health care costs.

## Introduction

Inflammatory bowel disease (IBD) is a group of disorders that cause sections of the gastrointestinal tract to become inflamed and ulcerated. IBD imposes a significant impact on the quality of life through ongoing symptoms, including reduced ability to work, social stigma, and restriction in career choices. IBD causes a great burden globally because of the direct costs of care and the indirect costs associated with disability and missed work [1-4]. In the United States, IBD ranks as 1 of the 5 most expensive gastrointestinal disorders despite it being the lowest in prevalence in the list of gastrointestinal disorders [5]. The total financial burden of IBD in the United States was estimated to be US $14.6 billion to US $31.6 billion in 2014 [6]; however, recent data have indicated that the total costs may far exceed these earlier estimates [7]. In Canada, wherein the prevalence of IBD is one of the highest in the world, the economic cost for IBD was conservatively estimated to be over US $0.9 billion in 2018 [8]. In Europe, 2.5-3 million people have been estimated to be affected with IBD with a direct health care cost of 4.6-5.6 billion Euros/year [9]. However, the treatment for IBD is not curative. Clinical management aims at inducing and maintaining remission by using pharmaceutical agents and surgery, and evolving treatment guidelines advocate rapid scale-up to biological agents for improving health outcomes and quality of life. Consequently, the health care costs are driven by the increasing use of biological agents—most importantly by anti-tumor necrosis factor-α therapy [10]. In China, retrospective analysis has shown an increasing incidence of IBD; it is estimated that the IBD prevalence in China, which includes up to 11.6 ulcerative colitis cases per 100,000 person-years and 1.4 Crohn disease cases per 100,000 person-years [11,12], is the highest in Asia [13].

China is facing a growing burden with the increased use of health care resources, including outpatient visits, emergency room visits, hospital admissions, and surgeries [14]. Medical insurance policies vary widely among provinces in China. It is important to understand the financial burden and health care access of Chinese patients with IBD to measure treatment values and optimize health care policies. However, the collection of accurate epidemiologic data in China has been hampered by the lack of a nationwide IBD registry. One study from China in 2017 reported that 30.6% of the patients with IBD spent over half of their income to cover medical costs [15]. A retrospective cohort study in Hongkong reported that the total direct medical expenditure was US $7,072,710 for 435 patients with IBD, wherein hospitalizations (33%) and 5-aminosalicylic acid (23%) therapy accounted for the most part [16]. However, economic data and association between financial burden and health care access in mainland China are still lacking.

The China Crohn’s & Colitis Foundation (CCCF) serves as a nonprofit, volunteer-driven organization dedicated to improving the quality of life of patients with IBD in China [14]. With the support of the CCCF platform, we sought to provide a national snapshot of the current status of care for patients with IBD through electronic questionnaires in 14 IBD centers in mainland China. The contents of our survey focused on health care expenditures and insurance, access to care and therapies, affordability, and financial stress related to IBD.

## Methods

### Survey Development and Delivery

**Figure 1:**
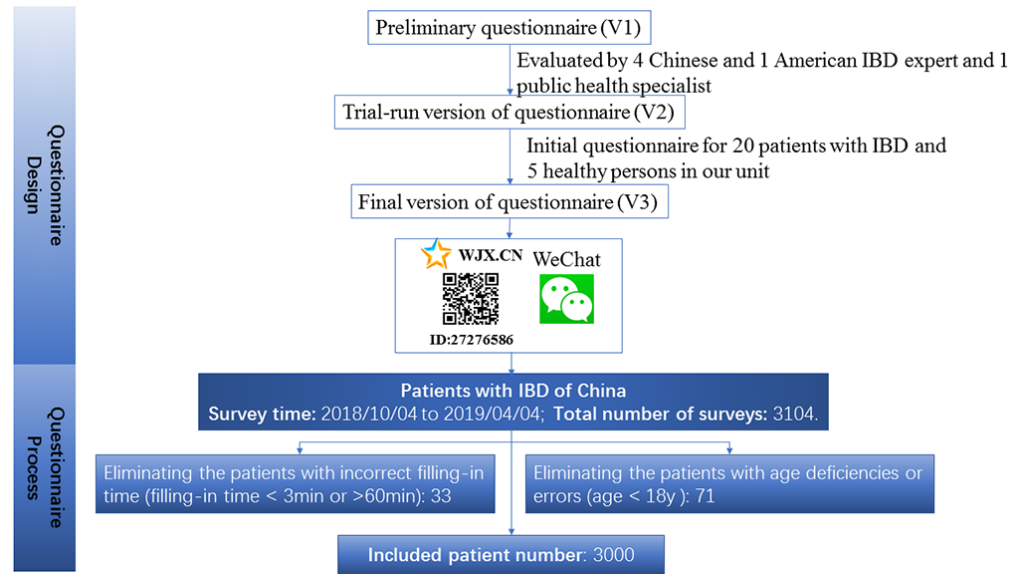
Flow chart depicting the selection of the survey sample for analysis.

We used a 64-item questionnaire developed by Rubin et al [1], which was modified from the IBD questionnaire and guidelines for the comprehensive intervention of chronic diseases in China to assess the financial burdens and health care access of patients with IBD in China. Our questionnaire focused on topics, including respondent disease type, disease severity, disease duration, IBD-related treatment, access to provider care, employment and insurance status, income, and strategies for affording care. As shown in Figure 1, the questionnaire was evaluated and modified by CCCF doctors from 14 IBD centers in mainland China and was produced by Wenjuanxing [17], which is a free and open platform for survey design. We delivered the web-based survey from October 4, 2018 to April 4, 2019 through the CCCF’s WeChat public platform*,* which is a popular source for patient education about IBD in China [18]. The inclusion criteria were informed consent, older than 18 years, and diagnosed with Crohn disease, ulcerative colitis, or indeterminate colitis. The questionnaires without age information were excluded. This study was approved by the medical ethics committee of the Second Affiliated Hospital, School of Medicine of Zhejiang University (No. 314).

### Clinical Variables

Demographic variables included IBD type (Crohn disease, ulcerative colitis, or indeterminate colitis), marital and work status, education level, income, and insurance status. Direct costs in this study mainly included outpatient costs, hospitalization costs, biological agent costs, and surgery costs. As biological agent costs were partly repeated with hospitalization costs, it was not calculated into the direct costs here. Indirect costs were defined as nonprescription medications, transportation costs, accommodation costs, etc; however, we only calculated the transportation and accommodation costs in our study. The clinical variables of indirect costs included transportation and accommodation costs. Variables of health care access included emergency room visit frequency, hospitalization duration, and waiting time for hospitalization.

### Statistical Analyses

Statistical analyses were conducted using SPSS software (version 25.0, IBM Corp). To estimate the average expenses, the mean and variance were calculated from the median value of the finite interval and the minimum value of the infinite interval. A binary logistic regression model was used to analyze the association between income or costs and variables of health care access.

## Results

### Population Characteristics

As shown in Figure 1, 3104 individuals from 14 IBD centers completed the questionnaire. Of those, 3000 respondents with a diagnosis of IBD were included for the analysis. Participants were eliminated from the data if they were younger than 18 years or did not provide age information (n=71) or if they completed the survey in less than 3 minutes or more than 60 minutes (n=33). The patients with IBD who completed the survey were distributed throughout all regions of mainland China. Most respondents were patients with Crohn disease (1922/3000, 64.1%), followed by those with ulcerative colitis (973/3000, 32.4%) or unclassified IBD (105/3000, 3.5%). Most respondents reported a disease duration of IBD of less than 5 years (2486/3000, 89.3%). The median age of the respondents was 34 years (range 18-73 years). Approximately 68.1% (2042/3000) of the patients were married (Table 1). The employment status of the patients with IBD was also assessed in our survey; 36.4% (1789/3000) of the patients had full-time work, 18.5% (555/3000) had part-time work, 6.9% (207/3000) were temporarily unemployed due to IBD, 20.7% (621/3000) were under the working age, 6.5% (196/3000) reported nonparticipation attributable to IBD, 6.7% (201/3000) were out of work, and 4.2% (127/3000) were retired.

**Table 1:** Background characteristics of the patients with inflammatory bowel disease (n=3000).

Characteristics		Value
**Sex, n (%)**		
	Female	1211 (40.4)
	Male	1789 (59.6)
**Inflammatory bowel disease type, n (%)**		
	Crohn disease	1922 (64.1)
	Ulcerative colitis	973 (32.4)
	Inflammatory bowel disease (unclassified)	105 (3.5)
**Inflammatory bowel disease duration (years), n (%)**		
	0-5	2486 (89.3)
	>5	514 (10.7)
Median age (range)		34 (18-73)
**Marital status, n (%)**		
	Married	2042 (68.1)
	Unmarried	958 (31.9)
**Work status, n (%)**		
	Working full-time	1093 (36.4)
	Working part-time	555 (18.5)
	Nonparticipation attributable to inflammatory bowel disease	207 (6.9)
	Underage	621 (20.7)
	Out of work	196 (6.5)
	Currently unemployed	201 (6.7)
	Retired	127 (4.2)
**Highest level of education attained, n (%)**		
	Illiterate	17 (0.6)
	Elementary/Junior/Senior school	1389 (46.3)
	College degree	1473 (49.1)
	Graduated or higher level education	121 (4.0)
**Income^a^ per month, n (%)**		
	<¥5000 (<US $723)	1490 (49.7)
	¥5000-¥9999 (US $723-$1445)	1073 (35.8)
	≥¥10,000 (≥US $1445)	437 (14.5)
**Insurance status, n (%)**		
	Uninsured	106 (3.5)
	Urban medical insurance	1568 (52.3)
	New type of rural cooperative medical care	1011 (33.7)
	Student health insurance	157 (5.2)
	Other medical insurance	158 (5.3)
**Commercial insurance, n (%)**		
	Yes	741 (24.7)
	No	2259 (75.3)
**Primary care provider, n (%)**		
	No primary care provider	1282 (42.7)
	Primary care provider	1718 (57.3)
	Inflammatory bowel disease expert^b^	1182 (68.8)

^a^US $1=¥6.9197.

^b^As identified by the patients.

### Financial Burden of IBD

The financial burden of IBD includes the direct and indirect costs incurred by individuals and society beyond the health care system. In estimation, the mean (SD) direct and indirect costs for per patient with IBD per year were conservatively ¥80,743.73 (¥54,973.12) (US $11,668.68 [$7944.44]) and ¥518.27 (¥1754.85) (US $74.90 [$253.60]), respectively. As shown in Table 2, for total outpatient costs, 41.7% (1252/3000) of the respondents with IBD reported spending more than ¥20,000 (US $2890) during the last 12 months. However, 46.5% of the patients (1394/3000) had no reimbursement for the outpatient costs. With respect to inpatient costs, 69.3% (2080/3000) of the patients with IBD reported paying less than ¥50,000 (US $7226) for hospitalizations during the last 12 months, while 30.7% (920/3000) of them paid more than ¥50,000 (US $7226) for their hospitalizations. The proportion of reimbursement for the hospitalization costs was more than 50% for 54.6% (1171/2143) of the patients with IBD. Among all the respondents, only 26.6% (799/3000) of the patients with IBD received more than 3 infusions of biological agents per year. However, 61.7% (626/1014) of them covered the costs of the biological agents at their own expense without any reimbursement. Moreover, 23.6% (707/3000) of the patients with IBD reported experiencing at least one surgery throughout their disease duration.

**Table 2:** Health care costs for inflammatory bowel disease treatment during the last 12 months.

Type of health care costs, expenses and reimbursements			n (%), Value
**Outpatient costs**			
	**Total outpatient expenses^a^ (n=3000)**		
		¥0-¥4999 (US $0-$722)	727 (24.3)
		¥5000-¥9999 (US $723-$867)	474 (15.8)
		¥10,000-¥19,999 (US $1445-$2890)	547 (18.2)
		over ¥20,000 (≥US $2890)	1252 (41.7)
	**Proportion of reimbursement** **(n=3000)**		
		Unknown	53 (1.8)
		0%	1394 (46.5)
		1%-49%	743 (24.7)
		50%-99%	801 (26.7)
		100%	9 (0.3)
**Hospitalization costs**			
	**Total hospitalization expenses (n=3000)**		
		0	857 (28.6)
		¥1-¥9999 (US $0-$1445)	268 (8.9)
		¥10,000-¥49,999 (US $1445-$7226)	955 (31.8)
		over ¥50,000 (≥US $7226)	920 (30.7)
	**Proportion of reimbursement (n=2143)**		
		0%	140 (6.5)
		1%-49%	832 (38.9)
		50%-99%	1169 (54.5)
		100%	2 (0.1)
**Biological agent costs**			
	**Frequency of use of biological agents (times) (n=3000)**		
		0	2072 (69.1)
		1-9	874 (29.1)
		Over 10	54 (1.8)
	**Proportion of reimbursement (n=1014)**		
		All at one’s own expense	626 (61.7)
		Partial reimbursement	378 (37.3)
		Complete reimbursement	10 (1.0)
**Surgery costs**			
	**Surgery costs (n=707)**		
		¥0-¥10,000 (US $0-$1445)	122 (17.3)
		¥10,001-¥20,000 (US $1445-$2890)	129 (18.2)
		¥20,001-¥49,999 (US $2890-$7226)	162 (22.9)
		over ¥50,000 (≥US $7226)	294 (41.6)
	**Proportion of reimbursement (n=707)**		
		0%	40 (5.7)
		1%-49%	295 (41.8)
		50%-99%	370 (52.3)
		100%	2 (0.3)

^a^US $1=¥6.9197.

The costs of the surgeries were more than ¥50,000 (US $7226) for 41.6% (294/707) of the patients, but only 52.3% (370/707) of these patients reported receiving reimbursements of over 50% during the last 12 months. Additionally, 76.4% (2292/3000) of the patients had dietitian costs, including nutrition powder, nutrition solutions, or a specific carbohydrate diet during the last 12 months. Most of them (1300/3000, 43.3%) reported paying less than ¥20,000 (US $2890) during the last 12 months. However, 56.3% (1290/2292) of those who had dietitian costs paid these costs at their own expense without any reimbursement. Of note, for those who underwent special treatments such as fecal bacteria transplantation and stem cell transplantation, 30.2% (152/475) of them reported receiving no reimbursement.

### Health Care Access

**Figure 2:**
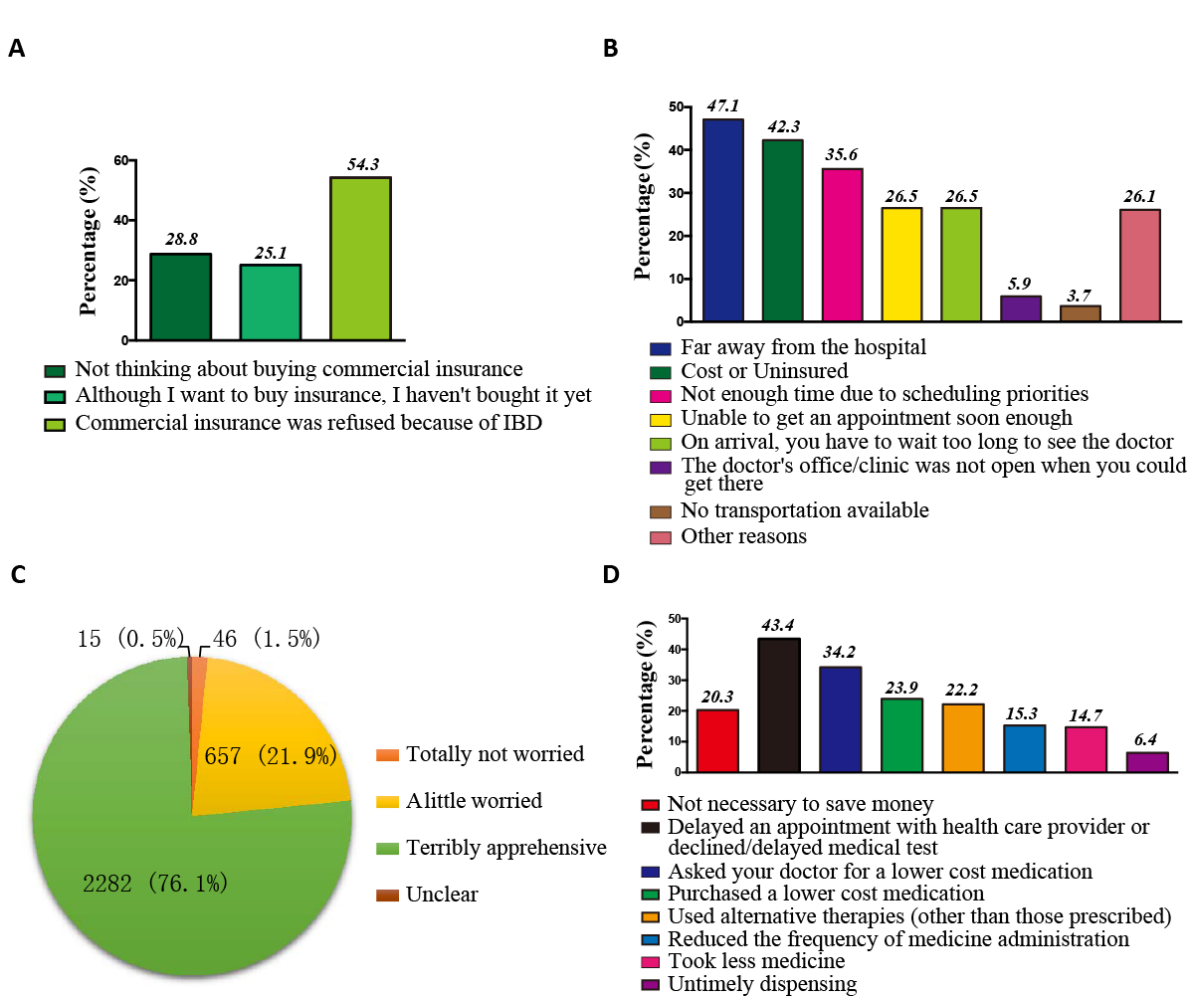
Analysis of the feelings of patients with inflammatory bowel disease (IBD) with regard to the health care and the associated costs. A. Reasons for patients with IBD to not have commercial insurance (n=3000). B. Reasons identified by patients with IBD for delaying health care (n=1389). C. Patients' anxiety about medical expenses (n=3000). D. How patients with IBD save money and delay care (n=3000).

We assessed the health care access of patients with IBD by mainly focusing on the health and life insurance, primary care providers, emergency room visits, and hospitalization utilization. The majority of the respondents (2894/3000, 96.5%) had regular medical insurance. However, 75.3% (2259/3000) of the respondents had no private commercial insurance; the reasons for patients being without private commercial insurance included that they had been refused coverage by an insurance company after their diagnosis of IBD (1399/2576, 54.3%), they had not thought about commercial insurance (741/2576, 28.8%), or they had not decided to buy it yet (647/2576, 25.1%) (Figure 2A). Approximately half of the respondents (1282/3000, 42.7%) had no primary care provider (Table 1). Of those who were identified with a primary care provider, 73.5% (1262/1718) of them had a gastroenterologist, 68.8% (1182/1718) had an IBD expert, 11.8% (203/1718) had an anorectal surgeon, 7.2% (123/1718) had a gynecologist, 2.7% (46/1718) had an abdominal surgeon, 1.0% (18/1718) had community physicians, and 0.6% (10/1718) had a family practitioner. Of note, some patients with IBD had more than one primary care provider. Moreover, 84.4% (2532/3000) of the patients with IBD were without IBD surgeons, and of the 15.6% (468/3000) of the patients with IBD who had IBD surgeons, only 60.9% (285/468) of their surgeons were considered full mastery. As shown in Table 3, we further assessed the experience of emergency room visits and found that 68.0% (2040/3000) of the total surveyed patients had visited an emergency room each year after IBD diagnosis: 52.2% (1567/3000) reported going to the emergency room 1 to 4 times per year and 15.8% (473/3000) reported going to the emergency room more than 5 times. Moreover, 74.8% (2243/3000) of the patients reported having experienced hospitalization, with 38.1% (1144/3000) of them reporting 1-3 occurrences, 31.5% (946/3000) reporting 4-10 occurrences, and 5.1% (153/3000) reporting more than 10 occurrences.

**Table 3:** Health care access of patients with inflammatory bowel disease (n=3000).

Health care access		n (%), Value
**Average emergency visits per year after IBD^a^**		
	0	960 (32.0)
	1-4 times	1567 (52.2)
	Over 5 times	473 (15.8)
**Hospitalization during last 12 months^b^**		
	0	757 (25.2)
	1-3 times	1144 (38.1)
	4-10 times	946 (31.5)
	over 10 times	153 (5.1)
**Average waiting time for hospitalization (days)**		
	1	443 (16.0)
	2-7	1547 (55.9)
	8-30	634 (22.9)
	Over 30	143 (5.2)
**Diagnosis and treatment of IBD in nearby medical institutions with nonspecialists in IBD treatment**		
	None	1411 (47.0)
	Often	689 (23.0)
	Once	220 (23.2)
	Occasionally	203 (6.8)
**Transportation facility for visiting the nearest IBD** **specialist**		
	Walking	22 (0.7)
	Bicycle	51 (1.7)
	Self-driving	420 (14.0)
	Public transport	1462 (48.7)
	Train	796 (26.5)
	Airplane	17 (0.6)
	Ship	1 (0.0)
	Others	231 (7.7)

^a^IBD: inflammatory bowel disease.

^b^Injections of biological agents or their dispensing are all performed in the hospitals.

The optimal treatment of IBD requires specialized health care; however, patients might travel a long distance to obtain care for IBD, and a greater distance to the referral health care center was demonstrated to be associated with an increased risk of needing IBD-related surgery and outcomes [16]. As shown in Table 4, 42.2% (1266/3000) of the patients covered a distance of more than 100 km, and 73.2% (2197/3000) of the patients needed more than 1 hour to reach the closest IBD center. The transportation cost for 62.4% (1871/3000) of the patients to the nearest IBD center was more than ¥100 (US $14), and the accommodation cost for 75.4% (2262/3000) of the patients to the nearest IBD center was more than ¥100 (US $14). Consequently, 53.0% (1589/3000) of the patients with IBD reported going to nearby medical institutions without IBD specialists. As shown in Figure 2B, there were several reasons for patients to delay health care, including being far away from the hospital (654/1389, 47.1%), the cost or being uninsured (587/1389, 42.3%), not having enough time due to scheduling priorities (494/1389, 35.6%), being unable to get an appointment soon enough (368/1389, 26.5%), and having to wait too long to see the doctor after arrival (368/1389, 26.5%). When asked about how they felt about the financial cost (Figure 2C), 98.5% (2954/3000) of the patients with IBD were worried about the medical expenses. Nearly 79.7% (2392/3000) of the patients with IBD sought ways to save money for their health care. As shown in Figure 2D, 79.7% (2392/3000) of the IBD patients sought ways to save money for their health care. Almost 43.3% of the patients (1303/3000) delayed an appointment with a health care provider or declined/delayed medical tests, 34.2% of the patients (1026/3000) asked doctors for a lower cost medication, and 22.2% of the patients (667/3000) used alternative therapies (other than those prescribed), while 30% of the patients (900/3000) even reduced the administration frequency or dosage of their medicine or 6.4% of the patients (192/3000) engaged in the untimely dispensing of their medication.

### Multifactor Analysis

We then focused on the influencing factors of emergency room visit times (Table 4). We found that patients with lower income (less than ¥5000 or US **$**723/month) were more likely to get into emergency rooms compared to patients with income more than ¥10,000 (US $1445) per month (odds ratio [OR] 1.947, 95% Cl 1.555-2.437; *P*<.001). Patients with lower transportation costs (¥0-¥99, US $0-$14) for visiting IBD specialists were less likely to visit emergency rooms compared to patients with higher transportation costs (>¥500, US $0-$72) (OR 0.622, 95% Cl 0.500-0.875; *P*=.004). Patients with ¥0-¥99 (US $0-$14) accommodation costs for visiting IBD specialists (OR 0.632, 95% Cl 0.494-0.810; *P*<.001) and ¥100-¥499 (US $14-$72) accommodation costs (OR 0.712, 95% Cl 0.584-0.866; *P*=.001) had less emergency room visit times than patients with >¥500 (US $72) accommodation costs (Table 4).

**Table 4:** Transportation costs and additional costs for medical treatments (n=3000).

Factor		n (%), Value	Emergency visit times		
			Odds ratio	95% Cl	*P* value
**Distance to the nearest medical institution with IBD^a^ specialists**					.63
	>100 km	1266 (42.2)	1	N/A^b^	N/A
	10-99 km	1145 (38.2)	1.106	0.896-1.365	.35
	<10 km	589 (19.6)	1.069	0.787-1.452	.67
**Income of patients (per month)**					*<.001*
	>¥10,000 (US $1445)	434 (14.5)	1	N/A	N/A
	¥5000-¥9999 (US **$**723-$1445)	1081 (36.0)	1.224	0.976-1.535	.08
	¥0-¥4999 (US $0-$723)	1485 (49.5)	1.947	1.555-2.437	*<.001*
**Time for visiting the nearest IBD specialist**					.73
	>6 hours	372 (12.4)	1	N/A	N/A
	1-5 hours	1825 (60.8)	1.105	0.844-1.447	.47
	<1 hour	803 (26.8)	1.060	0.745-1.508	.75
**Total transportation costs for visiting IBD specialists^c^**					*.02*
	>¥500 (US $72)	634 (21.1)	1	N/A	N/A
	¥100-¥499 (US $14-$72)	1234 (41.1)	0.816	0.650-1.024	.08
	¥0-¥99 (US $0-$14)	1132 (37.7)	0.622	0.500-0.875	*.004*
**Accommodation costs for visiting IBD specialists^c^**					*<.001*
	>¥500 (US $72)	791 (26.4)	1	N/A	N/A
	¥100-¥499 (US $14-$72)	1467 (48.9)	0.712	0.584-0.866	*.001*
	¥0-¥99 (US $0-$14)	742 (24.7)	0.632	0.494-0.810	*<.001*

^a^IBD: inflammatory bowel disease.

^b^N/A: not applicable.

^c^A multiple logistic regression model was used to obtain the *P* value. Values in italics are significant at *P*<.05.

## Discussion

In conjunction with large at-risk populations, the absolute number of patients with IBD in newly industrialized countries has the potential to approximate that in the western world by 2025 [3]. IBD places an economic strain on health systems due to expensive pharmaceutical therapy, the risk of hospitalization and surgery, and long-term monitoring. Our study provides a detailed description of the financial burdens and health care access for patients with IBD in China. We found that heavy financial burdens due to health care and travelling long distances to receive health care were the main problems faced by Chinese patients with IBD at present.

Our conservative data showed the mean (SD) direct and indirect costs for per patient with IBD per year were US $11,668.68 ($7944.44) and US $74.90 ($253.60) by estimation. Recent studies reported that in the United States, the lifetime financial burden of Crohn disease or ulcerative colitis per person was estimated at US $622,000 to US $405,000. The total financial burden for Crohn disease and ulcerative colitis in 2016 was US $498 billion and US $377 billion, respectively [19]. In Canada, the economic cost for IBD was estimated at over US $3520 per person per year in 2018. Moreover, for rheumatoid arthritis in China, the mean (SD) direct and indirect costs were US $1917.21 ($2559.06) and US $492.88 ($1739.74) per patient year [20]. This did not include the intangible costs such as out of study or work. These findings demonstrate that IBD incurs a heavier financial burden than other chronic disease such as rheumatoid arthritis in China, with similar cost status reported in other western countries.

Our study showed that in over one-third of the surveyed patients, the outpatient and inpatient expenses exceeded ¥20,000 and ¥50,000 (US $2890 and US $7226, respectively) during the last 12 months. Only one-third of the inpatient costs were reimbursed at a rate of 60%, while the reimbursement ratio was even lower for the outpatient expenses for most patients. In addition, more than half of the patients who received biological agent treatment reported paying for the biological agents by themselves, and nearly half of the surgical costs had a reimbursement rate of less than 50%. However, the income of 49.5% (1485/3000) of the patients with IBD was less than ¥5000 (US $722) per month. Moreover, IBD affects the prime working years of patients with IBD and potentially has considerable effects on employment. IBD-related work losses have received great attention worldwide [21]. As shown above, 1907 (63.5%) of the 3000 patients with IBD in our survey reported not being able to work full-time.

Medical insurance policies in China vary widely between different provinces and are also different between urban and rural areas. In our study, 96.5% (2894/3000) of the patients had health insurance, 33.7% (1011/3000) had coverage under a new type of rural cooperative medical care system, and 5.2% (157/3000) had student health insurance. This is due to the government’s universal coverage of basic medical insurance for citizens. In China, social health insurance programs generally consist of 3 programs: the rural New Cooperative Medical Scheme launched in 2003, the Urban Residents Basic Medical Insurance program launched in 2007, and the Urban Employee Basic Medical Insurance program launched in 1998 [22]. Although insurance programs have rapidly expanded during the past decade, the benefit packages and deductibles vary between the programs. Rural populations have more restricted access to health care than urban residents do, with a larger financial burden, mainly due to a lower funding level for the New Cooperative Medical Scheme, for which the coverage of outpatient services is almost nonexistent [16,22-24]. However, 75.3% (2259/3000) of the surveyed patients did not have commercial insurance, with 54.3% (1399/2576) of the patients having been refused commercial insurance because of IBD. IBD is a chronic disease and cannot be cured, and the purchase of commercial private insurance for patients with IBD is generally rejected in China. Therefore, IBD-related costs have become a heavy financial burden for patients.

We also found that 76.1% (2282/3000) of the patients with IBD reported experiencing tremendous apprehension due to medical expenses and seeking ways to save money, such as delaying an appointment with a health care provider or declining/delaying medical tests, asking their doctors for a lower cost medication, purchasing a lower cost medication, using alternative therapies (other than those prescribed), reducing the frequency of medicine administration, taking less medicine, or engaging in untimely dispensing. However, all these are very harmful approaches to disease management, which in turn may lead to disease recurrence and further aggravating the economic pressures.

In our study, 52.2% (1567/3000) of the patients reported needing to go to the emergency department 1-4 times per year because of IBD. Both transportation costs and accommodation costs for visiting IBD specialists were positively associated with emergency room visit times. One possible reason may be that those patients with lower income level saved money by decreasing the follow-up times, which might lead to disease status becoming uncontrollable. The other reason could be that patients with lower income or without commercial private insurance cannot afford such expensive but effective biological agents, which resulted in a lower level of remission. This is consistent with that reported previously that travelling long distances to obtain medical treatment for IBD hinders regular care and adversely affects the outcomes—especially increasing the risk of needing IBD-related surgery [25].

Effective measures for solving the economic problems are reducing the expenses and improving the disease status for IBD. The reimbursement ratio for IBD should be increased especially for outpatient expenses, which may be difficult for this large population. Moreover, IBD experts are very scarce, especially in the city community. Approximately 42.7% (1282/3000) of the patients with IBD were without a primary care provider, and gastroenterologists and IBD experts were the most important primary care providers. Patients with IBD have difficulty obtaining medical services in China because community physicians account for 1% of the primary care providers and family practitioners account for 0.6% of the primary care providers [26]. As a result, 28.3% of the patients reported engaging in emergency dispensing. Therefore, telemedicine might be a good solution. In 2020, one web-based study showed that the use and need of telemedicine have been increasing especially since the outbreak of COVID-19 [27]. In the same year, we performed a web-based research [28] about patterns of care for patients with IBD in China during COVID-19; the results showed that a quarter of the patients sought care via telemedicine with their IBD physicians. One randomized controlled study conducted by de Jong et al [29] compared the cost-effectiveness of telemedicine and standard care for management of IBD; they found that telemedicine was safe and reduced outpatient visits and hospital admissions compared with standard care. Del Hoyo et al [30] suggested that compared with standard and telephone care, telemedicine with web-based programs decreased the direct and indirect costs for patients with IBD; other studies by Elkjaer [31] and de Jong [32] have shown similar results. Mao et al [33] mentioned that CCCF has organized a group of volunteer gastroenterologists that specialize in IBD to offer web-based consultancy to patients with IBD. Another study [34] reported that they sent educational and instructional alerts and messages to web-based IBD groups of patients via WeChat and mailed the drugs to the patients who lived far from the hospital. Therefore, telemedicine was shown to be not only cost-saving but also a medium to provide more accurate disease information for patients with IBD, which will also improve their medical adherence.

This is the first large-scale nationwide patient survey to assess the financial burdens and health care access of patients with IBD in China. Our results reflect the current status of Chinese patients with IBD and are vital for the national policy design for patients with IBD and the planning of CCCF projects in the future. Moreover, it is vital for IBD doctors and nurses to know more about the specific situation of patients with IBD in China and to understand more about the behaviors of patients with IBD. Our study plays a significant role in strengthening the disease management and improving the quality of life of patients with IBD in China. The imperfect and inherent self-report survey methodology, including its reliance on respondents to provide accurate and bias-free responses, was one limitation of our study. However, the aim of this survey was to assess patients’ perception of access to health care and their financial burdens; thus, the self-report survey data are reasonable although they are imperfect. Moreover, patients with IBD who had limited access to the internet could not be included in our study, which was a potential source of selection bias in our study. While our study assessed patients with IBD in most regions of China, a large number of the included patients were mainly located in the provinces of Zhejiang, Anhui, and Jiangsu, wherein the economic status is higher than that in other areas; therefore, patients who could not access the internet may have worse economic status than our respondents. Thus, our study was just a mirror, reflecting part of the economic issues, but the reality is even worse than our estimation.

In conclusion, serious financial burdens and difficulties in health care access are the 2 major difficulties faced by patients with IBD in China. These problems need to be confronted by national health care systems, social force communities, and the patients themselves. Early purchase of private insurance and increasing the imbursement ratio of medical costs will cut the direct costs of IBD, and increasing use of telemedicine may be beneficial for decreasing both the direct and indirect costs for Chinese patients with IBD.

## Abbreviations

CCCF: China Crohn’s & Colitis Foundation
IBD: inflammatory bowel disease
OR: odds ratio
